# Effects of Milling Parameters on Residual Stress and Cutting Force

**DOI:** 10.3390/ma18163836

**Published:** 2025-08-15

**Authors:** Haili Jia, Wu Xiong, Aimin Wang, Long Wu

**Affiliations:** 1 Tianjin High-End Intelligent Machine Tool Engineering Research Center, Tianjin University of Technology and Education, Tianjin 300222, China; 17858925711@163.com; 2 School of Mechanical Engineering, Beijng Institute of Technology, Beijng 100081, China; wangam@bit.edu.cn; 3 School of Mechanical and Electrical Engineering, Shandong Jianzhu University, Jinan 250101, China; wulong0128@163.com

**Keywords:** 7075-T7451 aluminum alloy, surface residual stress, cutting forces, predictive modeling, milling parameters

## Abstract

The 7075-T7451 aluminum alloy, widely used in aerospace, aviation, and automotive fields for critical load-bearing components due to its excellent mechanical properties, suffers from residual stresses induced by thermo-mechanical coupling during milling, which deteriorate workpiece performance. This study explores how key milling parameters—spindle speed *n_c_*, feed per tooth *f_z_*, cutting depth *a_p_*, and cutting width *a_e_*—affect surface residual stress and cutting force via orthogonal experiments and finite element analysis (FEA). Results show *a_e_* is critical for X-direction residual stresses, while *f_z_* dominates Y-direction ones. Cutting force increases with *f_z_*, *a_p_*, and *a_e_* but decreases with higher *n_c_*. Multivariate regression-based prediction models for residual stress and cutting force were established, which effectively characterize parameter–response relationships with maximum prediction errors of 18.69% (residual stress) and 12.27% (cutting force), showing good engineering applicability. The findings provide theoretical and experimental foundations for multi-parameter optimization in aluminum alloy milling and residual stress/cutting force control, with satisfactory practical effectiveness.

## 1. Introduction

As a critical structural material in the aerospace industry, the 7075-T7451 aluminum alloy is widely used in load-bearing components due to its exceptional specific strength, corrosion resistance, and heat-treatment strengthening capability [1]. However, this alloy is prone to significant residual stresses during milling processes caused by non-uniform plastic deformation, leading to post-machining distortion and dimensional instability. In the fabrication of aerospace frames and beams, stress-relief-induced deformations often increase non-conformance rates and necessitate costly corrective processes. For instance, thin-walled beam structures typically exhibit flatness deviations of 0.1–0.5 mm after machining, directly compromising assembly accuracy. From a safety perspective, the 7075-T7451 is frequently employed in high-risk components such as aircraft landing gears and wing spars, where structural failure could result in catastrophic consequences. Industry statistics indicate that approximately 40% of failure cases in aerospace aluminum components are associated with machining-induced residual stress [2,3]. Therefore, the precise control of milling parameters to optimize residual stress distribution is crucial for enhancing the reliability of high-precision aluminum parts. This underscores the engineering necessity for a systematic investigation into this specific material.

The machining-induced residual stresses are predominantly attributed to the synergistic action of mechanical cutting forces and thermomechanical effects. The cutting force is strongly correlated with the cross-sectional area of the cut layer and is influenced by machining parameters such as $f_{z}$, $a_{e}$, and $a_{p}$. Meanwhile, temperature distribution is predominantly governed by cutting speed and cooling conditions. The surface compressive stress field is primarily generated through mechanical loading by cutting forces, whereas tensile stresses are thermally activated. Furthermore, stress redistribution occurs due to volume mismatch during phase transformations in the affected material zone. Based on these fundamental principles, the formation mechanisms of machining-induced residual stresses can be systematically categorized into two dominant theoretical models. In the mechanical-dominant theory, the surface compressive stress field is attributed to tool–workpiece interaction forces, with corresponding tensile stresses being induced in the substrate material as a mechanical equilibrium requirement. The thermal-mechanical model reveals that surface tensile stresses originate from inhibited thermal dilatation during machining, with compensating compressive stresses being generated in the bulk material to satisfy equilibrium conditions [4]. In practical machining operations, these two mechanisms typically interact synergistically. The dominant factor is ultimately determined by the relative intensity of mechanical-thermal effects under specific process conditions. As a critical indicator of surface integrity, residual stresses significantly influence component performance. Both the distribution characteristics and magnitude of residual stresses are directly correlated with key component performance metrics, including dimensional deformation, fatigue strength, and corrosion resistance. Generally, compressive stresses enhance material strength and fatigue-corrosion resistance while suppressing microcrack initiation. In contrast, tensile stresses tend to degrade fatigue performance and may induce detrimental effects such as intergranular corrosion [5].

The formation mechanisms of surface residual stresses during milling operations are inherently complex, involving multi-physics coupling effects. This complexity arises from three fundamental aspects: non-uniform plastic deformation, steep thermal gradients, and potential phase transformation processes. Permanent deformation initiates in the workpiece surface and subsurface layers once the stress exceeds the material’s yield strength. The resultant residual stresses are influenced by a complex interplay of multiple factors. These influencing factors primarily include machining parameters, tool geometric characteristics, and wear conditions, as well as workpiece material properties. Extensive research efforts have been devoted by numerous scholars to elucidating the influence mechanisms of various factors on residual stresses through diversified modeling approaches and advanced computational algorithms. From a methodological perspective, certain researchers have employed analytical modeling to investigate the coupled effects of complex influencing factors. Li et al. [6] developed a fuzzy inference model for machining systems comprising both rule-based and result-based frameworks. The model processes input parameters including linear choking degree, tool path trajectories, and cutting forces to generate outputs of machining-induced deformation values—using composite matching to measure the importance of uncertain inputs to outputs, thereby enabling the effective control of input uncertainties to significantly reduce the probability of machining deformation failures. Jiang et al. [7] developed an empirical superposition model for milling-induced residual stresses. The model effectively distinguishes the respective contribution ratios of milling forces versus thermal effects on residual stress generation. In studies addressing specific machining scenarios, Zheng et al. [8] proposed the concept of non-uniform milling. Predictions from the elasticity-derived model show excellent consistency with FEM simulations and experimental measurements, confirming its capability in characterizing residual stresses and machining deformations in milled aluminum alloy beam structures. Zhang et al. [9] developed an innovative methodology involving the milling of thin-walled specimens for deformation measurement. By calculating the mean value of MIRS (Machining-Induced Residual Stress), the deformation of thin-walled components can be predicted with experimental errors below 20%. Other researchers have concentrated their investigations on residual stress characteristics in specific materials and structural components. Li et al. [10] established a semi-analytical model for predicting bending deformations of aluminum alloy components induced by residual stresses. A systematic investigation was performed to elucidate the influence of residual stresses and T-section geometrical characteristics on deformation behavior, which was rigorously validated by coupled finite element analysis and experimental machining tests. Chen et al. [11] integrated Gray Relational Analysis, the Backpropagation Neural Network, and Non-dominated Sorting Genetic Algorithm-III into a unified analytical framework. The influencing factors of residual stresses in the milling processes of Mg-Li alloys were systematically investigated by them. The surface tensile and subsurface compressive stresses were found to have a typical spoon-shaped distribution, confirming the coupled effect of cutting forces and temperature. Jiang et al. [12] proposed a coupled modeling framework to characterize the interrelationships among cutting forces, thermal loads, and residual stress fields. The study concluded that tool geometry parameters and feed per tooth exert significant influences on cutting forces, which in turn dominantly affect the resultant residual stress profiles. The study by Sivam et al. [13] established the critical role of cutting speed in residual stress formation during the dry milling of the ZE41 magnesium alloy. Berry et al. [14] experimentally confirmed that increasing feed per tooth while decreasing cutting speed generates higher compressive stresses in the material’s surface layer. While improving fatigue life, this technique risks interfacial fracture initiation at the transition region separating deformed and undeformed material domains. The study by Matuszak et al. [15] demonstrated that cutting speed significantly affects tool wear and surface integrity characteristics (including residual stress) during the high-speed machining of the Ti-6Al-4V titanium alloy.

Zhang et al. [16] investigated the effects of cutting parameters on microstructural evolution and damage mechanisms during the micro-cutting of the 7075-T6 aluminum alloy. They analyzed the influences of cutting parameters on micro-cutting forces and surface topography via single-factor experiments, and established a 3D micro-cutting finite element model based on crystal plasticity theory to study residual stress, microstructural evolution, and damage behavior. The results indicate that the main cutting force first decreases and then increases with cutting speed, with the inflection point speed varying with cutting depth; an increase in cutting depth exacerbates microcrack propagation, while a deeper maximum residual compressive stress suppresses crack initiation. Yue et al. [17] proposed a residual stress profile prediction model integrating the exponential decay cosine (EDC) function, particle swarm optimization (PSO), and backpropagation (BP) neural network for the milling of the 7075-T6 aluminum alloy. They analyzed the key characteristics of residual stress profiles and conducted multi-objective optimization based on the Kriging model. Eventually, the constructed multi-objective optimization framework combined with NSGA-III, MOPSO, and TOPSIS algorithms enabled the acquisition of optimal cutting parameter combinations under different optimization modes. From an energy perspective, Zheng et al. [18] developed a machining-induced residual stress analysis model considering energy conversion in the milling process. They established a milling force prediction model, an effective cutting work model, a strain energy solution model, as well as prediction models for mechanical stress, thermal stress, and residual stress. The accuracy of these models was verified through milling experiments, showing that the milling force prediction error is approximately 5% and the residual stress prediction error is around 15%, indicating relatively small errors for both models.

Despite extensive research achievements, the fundamental understanding of residual stress formation mechanisms still faces significant theoretical challenges. Although the analytical method can reveal the basic laws of stress distribution, it is difficult to quantitatively describe the dynamic thermal—force coupling effect in the complex milling process [19]. The finite element simulation technique, while capable of modeling cutting processes, faces significant challenges in achieving efficient predictions owing to limitations in material constitutive model accuracy and computational resource requirements [20]. In contrast, the integrated approach combining finite element simulation, experimental validation, orthogonal design, and data-driven modeling facilitates the efficient quantification of process-residual stress relationships, offering a reliable foundation for manufacturing process optimization. However, most of the existing research focuses on the effect of a single parameter (e.g., cutting speed or feed) on residual stress, and the systematic research on the interaction of multiple parameters and the evolution of residual stress under finishing conditions is still insufficient. Therefore, this study investigates the influence of milling parameters on surface residual stresses in the 7075-T7451 aerospace aluminum alloy through a designed four-factor three-level orthogonal experiment. Meanwhile, X-ray diffraction (XRD) analysis is integrated with cutting force measurement technology to establish a comprehensive characterization system. A quantitative analysis is conducted to characterize residual stress distribution patterns and cutting force variations under different process parameters, followed by the development of a predictive model through statistical analysis methods. This study aims to provide theoretical foundations for optimizing milling processes of high-precision aluminum alloy components in aerospace applications, while offering experimental support for controlling residual stresses and cutting forces under multi-parameter interactions.

Based on the above research gaps, the core objectives of this study are as follows:

(1) The effects of spindle speed $n_{c}$, feed per tooth $f_{z}$, cutting depth $a_{p}$, and cutting width $a_{e}$ on residual stress (X/Y directions) and cutting force (X/Y directions) in the 7075-T7451 aluminum alloy were systematically investigated through a designed L9(3^4^) orthogonal experiment coupled with finite element simulation. Parameter significance was quantitatively ranked based on range analysis.
(2) Theoretical foundations and experimental support are provided for multi-parameter optimization in the milling process of the 7075-T751 aluminum alloy, enabling the precise control of residual stress distribution and cutting force magnitude. Through this approach, practical issues caused by machining-induced stresses—including poor dimensional stability and reduced fatigue life—can be effectively addressed in the production of aerospace critical components.
(3) A multiple regression-based predictive model for residual stress and cutting force was developed, with the parameter–response relationships quantitatively characterized. The model demonstrated prediction errors within the acceptable engineering threshold (≤20%), as validated through experimental measurements.

## 2. Materials and Methods

When simulating the milling process of aluminum alloys using finite element methods, it is essential to account for their inherent dynamic complexity. The commercially established finite element software ABAQUS 2023, with its Explicit solver module, is employed to handle the nonlinear dynamic computations inherent to the milling simulation. Additionally, the Dynamic, Temp-Displacement, Explicit analysis step is utilized to solve the thermo-mechanically coupled machining process. The primary simulation components consist of the milling cutter and aluminum alloy workpiece.

The 7075-T7451 alloy, a high-strength Al-Zn-Mg-Cu precipitation-hardened aluminum, exhibits outstanding mechanical properties and superior stress corrosion resistance, making it widely used in aerospace applications. Through specific heat treatment processes, the 7075 aluminum alloy can achieve exceptionally high strength characteristics, making it widely utilized in aerospace, automotive, and mechanical manufacturing sectors. It is particularly suitable for aircraft structures and other high-stress components demanding exceptional strength and corrosion resistance.

The primary chemical compositions of the 7075-T7451 aluminum alloy are presented in Table 1, while Table 2 displays its physical and mechanical properties (See Appendix A for details.). To enhance clarity, the units and sources for the detailed mechanical property indices and chemical compositions are provided below [21].

**Figure 1:**
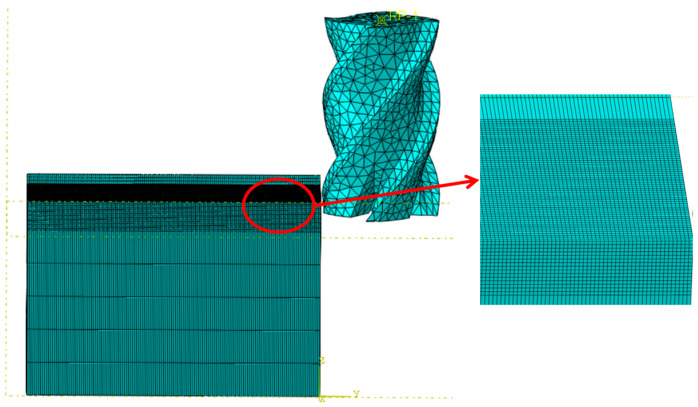
Simulation of milling process.

Figure 1 illustrates the simulated milling process of the aluminum alloy, in which a stationary workpiece is machined through the rotation and feed of the tool. The positions and directions of movement for both the workpiece and the tool on the machining center are simulated, with the nodes on the bottom surface of the workpiece being fully constrained. The milling cutter is modeled as a rigid body, maintaining its rotation along the Z-axis and allowing movement in the Y-direction. The feed is defined as the horizontal advance per revolution, while the rotation speed is referred to as the milling speed. The milling cutter used is a 4-flute carbide end mill, with the tool parameters detailed in Table 3. The workpiece dimensions are 70 mm × 40 mm × 40 mm. The initial temperature parameter is set to 20 °C, with the contact interface defined between the chip layer and the tool. The failure criterion for the milling process is surface failure. The Coulomb friction factor is established at 0.3, which is consistent with the parameter range recommended for aluminum alloy milling simulations in the existing studies. This value aligns with the tribological characteristics between cemented carbide tools and the 7075-T7451 aluminum alloy under dry cutting conditions, as validated by experimental studies on metal cutting interface behavior [22].

Additionally, controlling mesh density is essential for effective mesh division; a finer mesh yields higher computational accuracy. However, increasing mesh density directly leads to longer solution times and increased storage requirements. Therefore, it is crucial to optimize mesh sizing. To reduce calculation time and enhance result accuracy, we employ a local meshing technique for the workpiece. Specifically, in the cut section, particularly in areas of stress concentration and high plastic strain, the mesh is refined to a size of 0.05 mm. In non-critical regions, a coarser mesh is adopted with a size of 1 mm. The workpiece unit type is a hexahedral 8-node three-dimensional solid reduced integration element, C3D8RT. The cutting tool was modeled as a rigid body using four-node linear tetrahedral elements (C3D4) with a uniform mesh size of 0.3 mm.

In the model, the stress–strain relationship of the material significantly influences the prediction of the simulation results. Selecting an appropriate constitutive model to describe the material’s dynamic properties is crucial for establishing the finite element model. The milling process involves large strains and high temperatures, resulting in significant gradients in temperature and strain distribution. Therefore, selecting an appropriate intrinsic model based on the characteristics of the milling process is essential. Currently, the most commonly used constitutive models include Johnson–Cook (J-C), Bodner–Paton, and Follansbee–Kocks. The aluminum alloy 7075-T7451 was characterized using the Johnson–Cook (J-C) constitutive model, which effectively describes its thermo-mechanical behavior under large strains, high strain rates, and elevated temperatures.

The Johnson–Cook constitutive relationship can be formulated as [23]:

(1) $$\bar{\sigma }=\left(A+B{\bar{\varepsilon }}^{n}\right)\left[1+C\;ln\left(\frac{\dot{\bar{\varepsilon }}}{\dot{\bar{\varepsilon_{0}}}}\right)\right]\left(1-\frac{T-T_{0}}{T_{melt}-T_{0}}\right)$$

In this context, $\bar{\sigma }$ represents the equivalent stress; *A* denotes the yield strength; *B* indicates the strain hardening parameter; n refers to the hardening index; *C* signifies the basic parameter for strain rate reinforcement;$\;\bar{\varepsilon }$ represents the equivalent plastic strain per unit time; $\dot{\bar{\varepsilon }}$ denotes the equivalent strain rate; $\dot{\bar{\varepsilon_{0}}}$ indicates the equivalent reference strain rate; *T* refers to the actual temperature; $T_{0}$ is the reference temperature; and $T_{melt}$ represents the melting point temperature of the material.

Table 4 shows the Johnson–Cook constitutive model parameters for the aluminum alloy 7075-T7451.

The combination of the Johnson–Cook failure model and the Johnson–Cook constitutive model provides enhanced characterization of metal deformation failure under large strains, and thus it is adopted. The Johnson–Cook failure model defines the damage parameter based on the equivalent plastic strain at element integration points, expressed as

(2) $$D=\sum \frac{\Delta {\bar{\varepsilon }}^{pl}}{{\bar{\varepsilon }}_{f}^{pl}}$$

where $\Delta {\bar{\varepsilon }}^{pl}$ represents the equivalent plastic strain increment, and ${\bar{\varepsilon }}_{f}^{pl}$ denotes the failure strain, and is defined as

(3) $${\bar{\varepsilon }}_{f}^{pl}=\left[d_{1}+d_{2}exp\left(d_{3}\frac{p}{q}\right)\right]\left[1+d_{4}\ln\left(\frac{\dot{\bar{\varepsilon }}}{\dot{\bar{\varepsilon_{0}}}}\right)\right]\left(1+d_{5}\frac{T-T_{0}}{T_{melt}-T_{0}}\right)$$

The hydrostatic pressure to bias stress ratio is p/q (where q denotes the shear stress, i.e., the von Mises equivalent stress), and the temperature term together with the shear failure parameters are $d_{1}\;\mathrm{to}\;d_{5}$ defined in the Johnson–Cook failure model. The damage parameter *D* is accumulated at the end of each incremental analysis step. Material failure occurs when *D* exceeds 1, at which point the element is deleted. The Johnson–Cook shear failure parameters for the 7075-T7451 aluminum alloy are presented in Table 5 [22].

**Figure 2:**
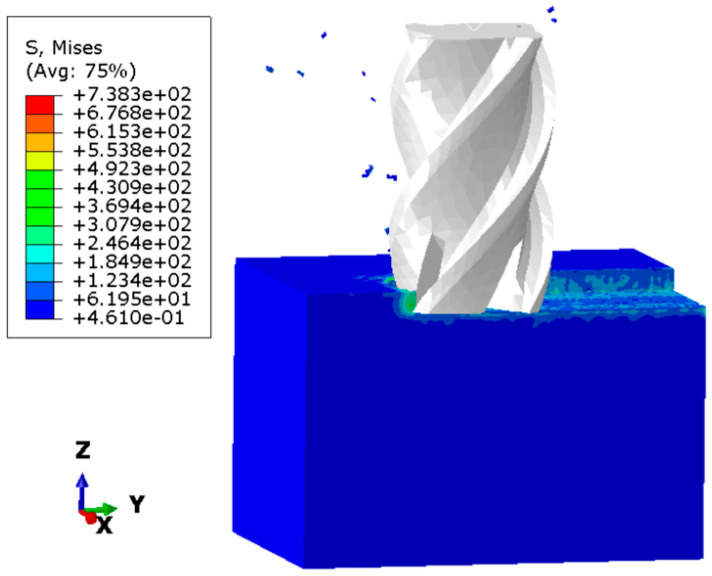
Simulation cloud diagram of the milling process.

To validate the established finite element model for milling process simulation, three distinct cutting parameter combinations, as listed in Table 6, were selected for numerical verification. Figure 2 displays the simulated milling process cloud diagram, while the predicted residual stresses are reported for both the feed direction (Y direction) and its perpendicular direction (X direction).

**Figure 3:**
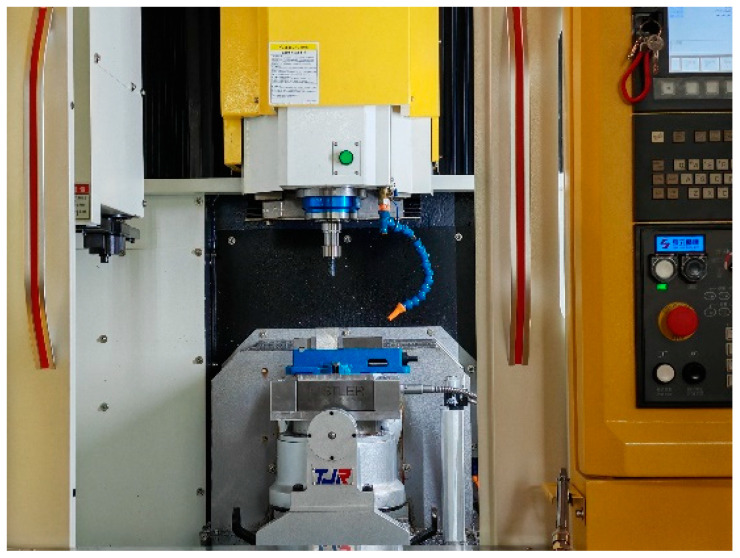
FLM540V precision machining center.

**Figure 4:**
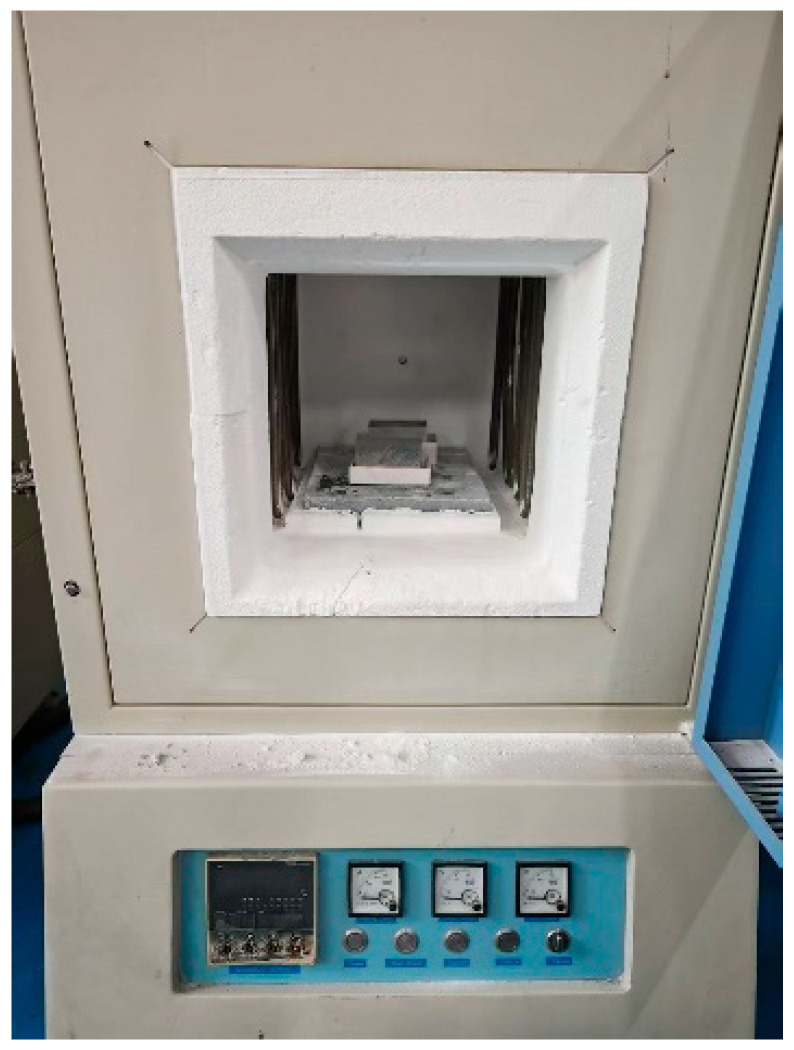
GF12Q-III Box-type Muffle Furnace.

**Figure 5:**
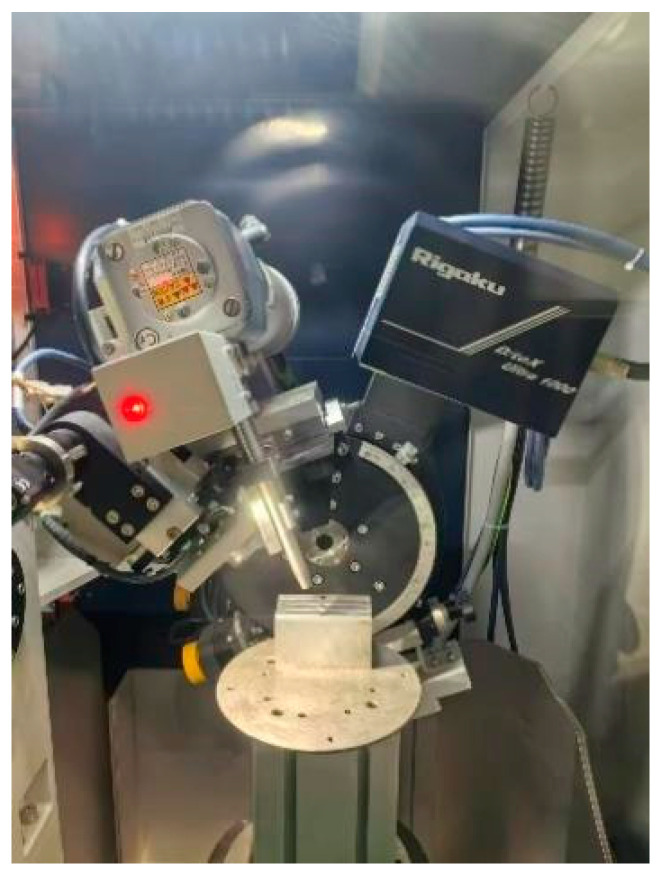
Auto MATE Ⅱ stress analyzer.

**Figure 6:**
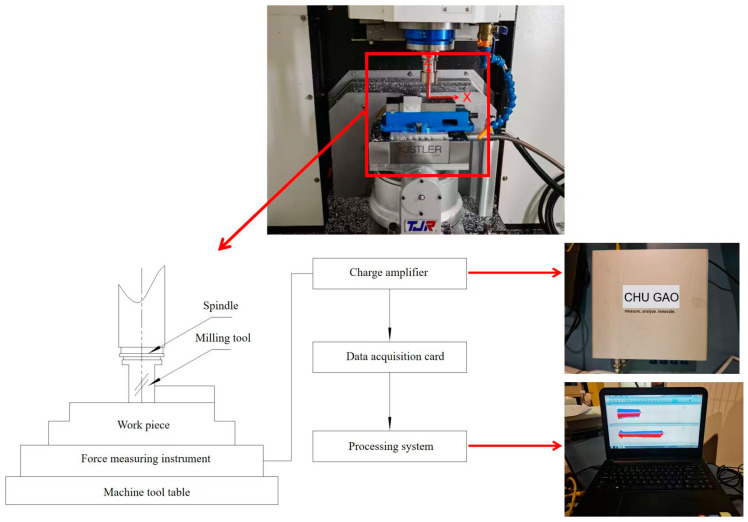
Kistler 9114B force measurement system.

This experiment was conducted at an FLM540V precision machining center with XYZ axis travels of 400 mm, 400 mm, and 300 mm, respectively, and a maximum spindle speed of 9000 rpm, as illustrated in Figure 3. The tools and workpieces employed in the experiments were consistent with the simulation model. All milling experiments were conducted using brand-new carbide end mills (parameters listed in Table 3) to ensure consistent initial cutting edge sharpness. A fresh tool was employed for each orthogonal test group to prevent the potential influence of edge wear caused by prolonged cutting on result stability. To mitigate pre-existing residual stress effects, all test coupons were subjected to a standardized annealing process before machining operations. A chamber muffle furnace was utilized to anneal the specimen blocks (Model: GF12Q-III, Manufacturer: Mafulu, Country of Origin: Tianjin, China, effective heating zone dimensions of the furnace chamber: 300mm × 250mm × 250 mm, maximum operating temperature: 1200 °C, temperature control accuracy: ±1 °C), as illustrated in Figure 4. The annealing temperature was maintained at 260 ± 5 °C for 2 h, followed by cooling to room temperature within the furnace. Following specimen preparation, residual stresses on the milled surfaces were measured using an Auto MATE Ⅱ stress analyzer (Model: AutoMATE II, Brand: Rigaku, Country of Origin: Tokyo, Japan, Detection Accuracy: ±5 MPa, 2θ Scanning Range: 98–168°, Maximum Power: 3 kW), with the measurement procedure detailed in Figure 5. For the reliable evaluation of milling-induced residual stress profiles, a tri-point sampling strategy was implemented across each machined layer, where the arithmetic mean of the spatially distributed measurements was adopted as the characteristic residual stress value. The cutting force was synchronously collected by a three-axis piezoelectric force sensor (Kistler 9114B, Kistler, Winterthur, Switzerland) at a sampling rate of 10,000 Hz, as depicted in Figure 6.

## 3. Results

This chapter presents the key findings of this study, focusing on model validation and orthogonal experimental analysis. First, the validity and accuracy of the established finite element model for the milling process were verified by comparing finite element simulation results with experimentally measured data, followed by error analysis. Subsequently, based on a four-factor, three-level orthogonal experimental design, the predictive modeling of surface residual stress and cutting forces was conducted, and empirical formulas were derived through regression analysis. Finally, the developed predictive models were validated, and their prediction accuracy was evaluated, laying the foundation for the subsequent analysis of the influence of milling parameters on residual stress and cutting forces.

### 3.1. Experimental Validation and Error Analysis

Table 7 summarizes the comparative results between the simulated and experimentally measured surface residual stresses and cutting forces in both X and Y directions.

Table 7 presents the maximum and average relative errors between simulated and experimental results for surface residual stresses: 16.48% and 13.98% in the X-direction, and 11.86% and 8.71% in the Y-direction. For milling forces, the relative errors are 12.11% and 9.02% in the X-direction, while those in the Y-direction are 11.62% and 8.47%. Experimental results confirm the validity and accuracy of the proposed milling process finite element simulation model.

### 3.2. Modeling of Surface Residual Stress and Cutting Force Prediction

The $n_{c}$, $a_{p}$, $a_{e}$, and $f_{z}$ were selected as parameters to design a four-factor, three-level (L9($3^{4}$)) orthogonal test. This study focuses on the milling residual stresses in the component’s surface layer after the final finishing process. The primary factors influencing process parameters include the machining characteristics, experimental machine tool, and cutting tool performance. The milling parameters were set to the following: spindle speed $n_{c}$ levels are set at 3000 r/min, 4000 r/min and 5000 r/min; $f_{z}$ levels are 0.01 mm/z, 0.02 mm/z, and 0.03 mm/z; $a_{p}$ levels are 0.5 mm, 1 mm, and 1.5 mm; and $a_{e}$ levels are 6 mm, 9 mm, and 12 mm. Table 8 presents the orthogonal experimental groups.

**Figure 7:**
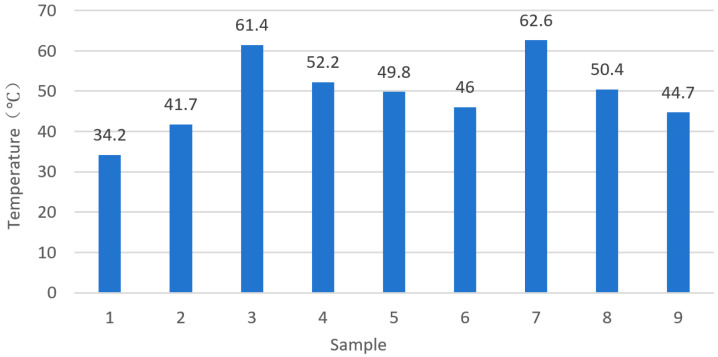
Maximum temperature distribution in milling simulation.

From the distribution of the maximum temperature in the milling simulation of the orthogonal groups shown in Figure 7, it can be seen that as the spindle speed and feed rate increase in the orthogonal experiment, the cutting temperature shows a significant upward trend. The maximum temperature reaches 62.6 °C (Simple 7), and the minimum temperature is 34.2 °C (Simple 1). The temperature difference between the groups exceeds 28 °C, which intuitively reflects the complexity of the thermo-mechanical coupling effect in milling. From the perspective of the thermo-mechanical coupling physical mechanism, combined with the residual stress test results (Table 9), the stress generation logic of groups with different temperature characteristics can be analyzed as follows:

(1) High temperature-dominated group (taking Simple 7 as an example): The cutting temperature of 62.6 °C makes the thermal expansion effect of the workpiece surface material significant. After the material in the high-temperature region undergoes thermal expansion, it is constrained by the surrounding undeformed matrix. During the cooling and contraction process, the thermal expansion inhibition effect dominates the generation of residual stress. The experimentally measured Y-direction residual stress of this group reaches −129.4 MPa, which highly coincides with the theoretical mechanism in the thermo-mechanical coupling model that “the contribution ratio of thermal stress increases with the rise in temperature”, verifying the prediction logic of the model for thermal expansion-induced stress.
(2) Mechanical force-dominated group (taking Simple 1 as an example): At 34.2 °C, the thermal effect is relatively weak, and mechanical loads such as cutting force and friction force between the tool and the workpiece become the main inducements for residual stress. At this time, the thermal expansion deformation of the material is small, and the plastic deformation caused by mechanical loads becomes the main cause of stress generation, which is consistent with the assumption in the model that “under the working condition of low heat input, mechanical force dominates the evolution of residual stress”.

Extensive milling residual stress data were obtained through orthogonal experiments (Table 9), enabling the derivation of an empirical calculation formula via mathematical processing. Regression analysis, a statistical method for investigating variable correlations, was applied to examine the four influencing factors in the orthogonal experiment. Given the prevalence of exponential empirical formulas in metal cutting, Equation (4) adopts this form to characterize the relationship between the milling parameters and residual stresses:

(4) $$\sigma =K_{t}\cdot {n_{c}}^{a_{1}}\cdot {f_{z}}^{a_{2}}\cdot {a_{p}}^{a_{3}}\cdot {a_{e}}^{a_{4}}$$

In this context,${\;K}_{t}$ represents a coefficient associated with cutting conditions.

This value can be derived by applying the natural logarithm

(5) $$ln\sigma =lnK_{t}+a_{1}lnn_{c}+a_{2}lnf_{z}+a_{3}lna_{p}+a_{4}lna_{e}$$

Simplify

(6) $$y=a_{0}+a_{1}x_{1}+a_{2}x_{2}+a_{3}x_{3}+a_{4}x_{4}$$

This yields a linear equation, indicating that the independent variables $x_{1}$, $x_{2}$, $x_{3},$ and $x_{4}$ exhibit linear relationships with the dependent variable y.

The empirical regression equations were derived through linear regression analysis using MATLAB R2022a’s regress function, as presented in Equations (7) and (8).

(7) $$\sigma_{x}=-24.108\cdot {n_{c}}^{0.031}\cdot {f_{z}}^{-0.383}\cdot {a_{p}}^{-0.096}\cdot {a_{e}}^{-0.67}$$

(8) $$\sigma_{y}=-10.996\cdot {n_{c}}^{-0.091}\cdot {f_{z}}^{-0.832}\cdot {a_{p}}^{0.23}\cdot {a_{e}}^{-0.322}$$

The cutting force empirical formula was subsequently derived using the aforementioned methodology, as expressed in Equations (9) and (10):

(9) $$F_{x}=10.61\cdot {n_{c}}^{0.297}\cdot {f_{z}}^{0.62}\cdot {a_{p}}^{0.882}\cdot {a_{e}}^{0.403}$$

(10) $$F_{y}=105.45\cdot {n_{c}}^{0.01}\cdot {f_{z}}^{0.389}\cdot {a_{p}}^{0.584}\cdot {a_{e}}^{0.16}$$

### 3.3. Validation of Surface Residual Stress and Milling Force Prediction Models

During the model validation phase, three additional experimental datasets were employed to evaluate the prediction accuracy through comparative analysis between the numerical results and experimental measurements (Table 10 and Table 11).

Data summary: The maximum error and average error of the surface residual stress prediction model in the X-direction are 15.48% and 13.55%, respectively, while the maximum error and average error in the Y-direction are 18.69% and 16.16%, respectively.

Data summary: The maximum error and average error of the cutting force prediction model in the X-direction are 9.01% and 7.27%, respectively, while the maximum error and average error in the Y-direction are 12.27% and 10.96%, respectively.

Yue et al. [17] proposed an EDC-PSO-BP neural network model trained on 25 experimental datasets. This model achieved a correlation coefficient of 96.0–98.9% in predicting residual stresses during the milling of the 7075-T6 aluminum alloy, with a maximum error of only 7.42%, indicating strong nonlinear fitting capabilities. However, its performance relies on large sample sizes and suffers from “black-box” limitations. In contrast, Zheng et al. [18] developed a purely physics-based model derived from energy conversion and thermo-mechanical coupling theory. Their model yielded milling force prediction errors of ≤10% and residual stress errors of approximately 15%. Although the underlying mechanism is well-defined, the model demands accurate material parameters and computationally expensive iterative simulations.

In this study, the multiple regression model, constructed from only 9 orthogonal experimental sets, exhibits maximum prediction errors of 18.69% for residual stress and 12.27% for cutting force. Although slightly less accurate, the proposed regression model provides clear physical interpretability and computational efficiency, rendering it more practical for small-sample engineering applications. These three approaches thus form a complementary framework, balancing prediction accuracy, mechanistic transparency, and computational cost.

## 4. Discussion

**Figure 8:**
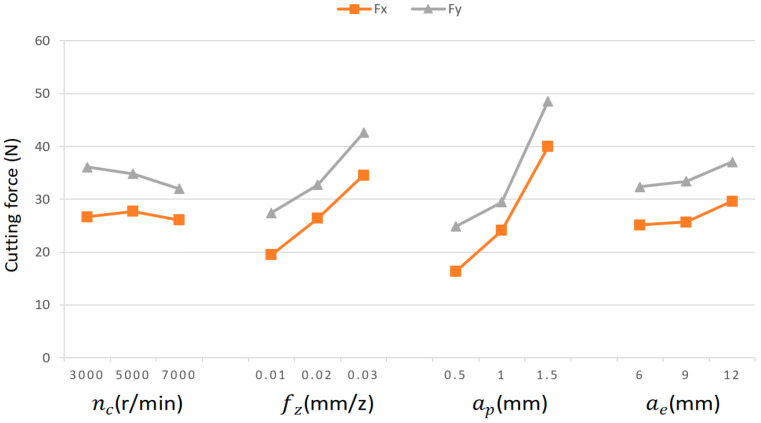
Trend of cutting forces with variation in cutting parameter levels.

Range analysis was performed on the orthogonal experimental data to systematically investigate the influence of the milling parameters on surface residual stress and cutting force characteristics. The range values of the parameter effects on cutting forces are presented in Table 12. Figure 7 shows the cutting forces variations under different parameter levels. The cutting forces Fx and Fy increase with the higher levels of $f_{z}$, $a_{p},$ and $a_{e}$ within the tested range. Analysis of the underlying mechanisms reveals that increases in parameters such as $f_{z}$, $a_{p}$, and $a_{e}$ directly lead to an enlargement of the undeformed chip thickness and instantaneous cutting area. As a high-strength precipitation-hardened aluminum alloy, the 7075-T7451 exhibits high yield strength and work-hardening behavior, which necessitate higher cutting forces to overcome the material’s resistance to plastic deformation and achieve effective shearing. Among these parameters, $a_{p}$ exerts the most significant influence, as it directly determines the axial depth involved in the cutting process, thereby substantially increasing the material removal rate and the contact area between the tool and the workpiece. Experimental observations demonstrate a significant decreasing trend in cutting force with increasing spindle speed within the tested parameter range. This phenomenon can be attributed to the enhanced thermal softening effect of the workpiece material at higher cutting speeds, coupled with reduced work hardening. This is attributed to the significant thermal softening behavior of the 7075-T7451 aluminum alloy at elevated temperatures: its flow stress decreases with increasing temperature, which facilitates easier material shearing and consequently reduces the cutting force. The synergistic effect of these two mechanisms leads to the observed cutting force reduction. The parameter influence magnitude follows: $a_{p}$ > $f_{z}$ > $a_{e}$ > $n_{c}$. These results show a strong consistency with the empirical cutting force equations. Figure 8 illustrates the milling force trends under different parameter levels.

**Figure 9:**
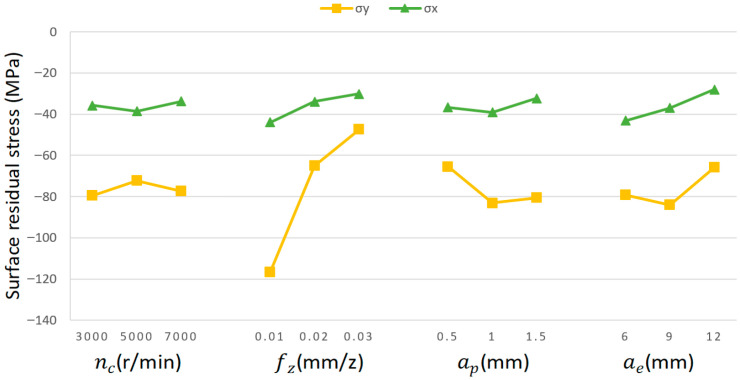
Trend of residual stresses with variation in cutting parameter levels.

As shown by the range analysis in Table 12, within the predetermined milling parameter range, $a_{e}\;$exhibits the most pronounced influence on X-directional residual stresses. Followed by $f_{z}$, and $a_{p}$—$n_{c}$ has the least effect. The underlying analysis reveals that expanding $a_{e}$ extends the effective range of cutting force application. The expansion of the cutting force-affected zone enlarges both the scope and intensity of plastic deformation, consequently leading to elevated residual stress levels. Furthermore, $a_{e}\;$significantly influences the distribution and dissipation of machining-induced heat, thereby altering the thermomechanical coupling state of the material and ultimately affecting the resultant residual stress characteristics. Based on the results presented in Table 12, within the defined milling parameter range, the ranking of cutting parameters’ influence on Y-directional residual stresses is as follows: $f_{z}$ > $a_{e}$ > $a_{p}$ > $n_{c}$. This is due to the fact that $f_{z}$ acts directly on the thickness of the cutting layer. The Y-direction residual stress is predominantly governed by $f_{z}$, which determines the undeformed chip thickness and consequently controls both the normal cutting force intensity and plowing effect depth. This mechanism significantly enhances the extent of plastic deformation within the material. Therefore, during subsequent process optimization, prioritizing the adjustment of $a_{e}$ and $f_{z}$ is essential for achieving effective control over X-directional residual stresses. For Y-directional residual stress control, primary optimization efforts should focus on $f_{z}$ as the dominant adjustment parameter. Figure 9 illustrates the residual stress trends across parameter levels.

Although orthogonal experimental design is primarily used to evaluate the main effects of the parameters, its balanced dispersion still provides the possibility for a simplified analysis of the interaction between key parameter pairs. To analyze the interactive influence of $f_{z}$ and $a_{p}$ on cutting force, samples 1, 2, and 3 with a spindle speed of 3000 r/min (Table 8) were selected to weaken the interference of $n_{c}$. The results show that when $f_{z}$ increases from 0.01 mm/z to 0.02 mm/z and $a_{p}$ increases from 0.5 mm to 1 mm, the X-direction cutting force ($F_{x}$) rises from 7.19 N to 22.41 N, with an increase rate of 212%. However, when $f_{z}$ further increases to 0.03 mm/z and $a_{p}$ increases to 1.5 mm, the increase rate of $F_{x}$ decreases to 125%. This indicates that the influence of $a_{p}$ on cutting force weakens with the increase of $f_{z}$: at low $f_{z}$, the effect of $a_{p}$ in expanding the axial cutting area is more significant; at high $f_{z}$, the thickness of the cutting layer dominates material removal, and the marginal effect of $a_{p}$ is weakened, reflecting the interaction between the two parameters.

To analyze the interactive influence of $a_{e}$ and $f_{z}$ on residual stress, samples 2, 4, and 9 with a constant cutting depth of 1 mm (Table 8) were selected to control the interference of $a_{p}$. The results show that when $a_{e}$ increases from 6 mm to 9 mm and $f_{z}$ decreases from 0.03 mm/z to 0.02 mm/z, the absolute value of $\sigma_{Y}$ rises from 57.91 MPa to 82.60 MPa, with an increase rate of 43%. However, when $a_{e}$ further increases to 12 mm and $f_{z}$ decreases to 0.01 mm/z, the increase rate of the absolute value of $\sigma_{Y}$ drops to 32%. It can be seen that the increase in $a_{e}$ weakens the influence of $f_{z}$: at small $a_{e}$, the cutting area is concentrated, and the enhancement of plastic deformation caused by the decrease in $f_{z}$ is more significant; at large $a_{e}$, the deformation energy is dispersed, and the regulatory effect of $f_{z}$ is diluted, confirming the interactive effect between the two parameters.

It should be explicitly noted that the simplified analysis in this study based on the L9(3^4^) orthogonal matrix has limitations. On one hand, in the selected “fixed secondary parameter groups”, there are still differences in other parameters, which may cause slight interference to the interaction trends. On the other hand, the 9 sets of experimental data cannot cover all level combinations of the parameters, making it difficult to quantify the precise intensity of the interaction effects. Future research can be further verified through full-factor experiments or response surface methodology.

## 5. Conclusions

This study systematically investigates the milling of the 7075-T7451 aluminum alloy through integrated finite element simulation and orthogonal testing, yielding the following key conclusions regarding residual stress and cutting force distribution patterns and predictive modeling:

(1) Comparative analysis between the simulation and experimental data reveals maximum relative errors of 16.48% (X-directional) and 11.86% (Y-directional) for surface residual stresses, with mean errors of 13.98% and 8.71%, respectively. For the cutting force, the maximum relative errors reach 12.11% (X-directional) and 11.62% (Y-directional), with mean errors of 9.02% and 8.47%. These results demonstrate that the developed dynamic thermomechanical coupling model accurately characterizes residual stresses and cutting force distributions during milling.
(2) The influence laws of the process parameters on cutting forces in the X- and Y-directions are summarized as follows: $a_{p}$ is the dominant factor, followed by $f_{z}$ and $a_{e}$. Cutting forces exhibit a positive correlation with $f_{z}$, $a_{e},$ and $a_{p}$, whereas an inverse trend is observed for $n_{c}$ due to thermal softening. Therefore, optimizing cutting forces requires prioritizing $a_{p}$ control, complemented by an elevated $n_{c}$ to exploit thermal softening for force suppression.
(3) Range analysis indicates that $a_{e}$ contributes most significantly to X-direction residual stresses, followed by $f_{z}$ and $a_{p}$, while $n_{c}$ shows negligible effects. For Y-direction residual stresses, $f_{z}$ dominates as the primary influencing factor, with $a_{e}$ and $a_{p}$ being secondary contributors. Therefore, subsequent process optimization should prioritize $a_{e}$ and $f_{z}$ adjustment for X-direction residual stress control, whereas $f_{z}$ requires particular attention when reducing Y-direction residual stresses.
(4) An exponential predictive model relating residual stress to cutting forces was developed through multivariate regression analysis. During model validation, the maximum prediction errors reached 18.69% for residual stress and 12.27% for cutting force, indicating the robust engineering applicability of the proposed model.

Future studies could further investigate the influence of milling parameters on other high-performance alloys (e.g., 2024 aluminum alloy, titanium alloys) or explore the mechanisms of tool coatings and wear effects on surface quality and residual stress, thereby providing support for optimizing the machining of a broader range of materials.

It should be noted that this study utilized the chemical composition and mechanical properties data of the 7075-T7451 aluminum alloy reported in the existing literature (Reference 21), without conducting independent material characterization. While these reference data are widely accepted in the field, we acknowledge that potential variations in material composition (even within the specification limits) may influence residual stress formation and cutting force characteristics. This constitutes a limitation of the present study. Future research should incorporate the following: (1) comprehensive material characterization including chemical composition analysis and mechanical testing prior to machining experiments; (2) a systematic investigation of how compositional variations within the 7075-T7451 specifications affect machining-induced residual stresses.

## Figures and Tables

**Table 1:** The 7075-T7451 aluminum alloy main chemical composition.

Element	Zn	Mg	Cu	Fe	Si	Mn	Cr	Ti	Al
Percent/%	5.1	2.1	1.2	0.5	0.4	0.3	0.18	0.2	tolerance

**Table 2:** Mechanical properties of 7075-T7451 aluminum alloy.

Mechanical Properties	$\sigma_{s}$ (MPa)	$\sigma_{b}$ (MPa)	ρ$\;(\mathrm{kg}/m^{3}$)	E (GPa)	δ (%)	HBW
Values	455	524	2850	70.3	11%	150

**Table 3:** Milling cutter parameters.

Tool Material	Cemented Carbide
Cutting edge diameter	12 (mm)
Cutting edge length	30 (mm)
Flute count	4
Helix angle	45°
Overall length	75 (mm)

**Table 4:** Johnson–Cook model parameters for 7075-T7451 aluminum alloy.

*A* (MPa)	*B* (MPa)	*C*	*n*	*m*	$T_{melt}$ (K)	$T_{0}$ (K)
435	278.94	0.019	0.34	0.96	1008	293

**Table 5:** Johnson–Cook shear failure parameters for 7075-T7451 aluminum alloy.

$d_{1}$	$d_{2}$	$d_{3}$	$d_{4}$	$d_{5}$
0.071	1.248	−1.142	0.147	0.01

**Table 6:** Milling parameters.

Sample	$n_{c}$ (r/min)	$f_{z}$ (mm/z)	$a_{p}$ (mm)	$a_{e}$ (mm)
1	3000	0.03	1.5	12
2	5000	0.03	0.5	9
3	7000	0.01	1.5	9

**Table 7:** Comparison of simulation and experimental results for residual stress and cutting force.

Sample	X Residual Stress (MPa)			X Cutting Force (N)			Y Residual Stress (MPa)			Y Cutting Force (N)		
	$\mathrm{Sim}.\sigma_{X-s}$	Exp. $\sigma_{X-e}$	Error (%)	Sim. $F_{X-s}$	Exp. $F_{X-e}$	Error (%)	Sim. $\sigma_{Y-s}$	Exp. $\sigma_{Y-e}$	Error (%)	$\mathrm{Sim}.F_{Y-s}$	$\mathrm{Exp}.F_{Y-e}$	Error (%)
1	−18.24	−21.84	16.48	50.38	45.71	10.22	−44.41	−48.60	9.45	61.47	65.49	6.78
2	−34.23	−39.45	13.23	23.81	27.09	12.11	−40.21	−45.62	11.86	32.86	37.18	11.62
3	−38.84	−44.26	12.25	30.79	29.40	4.72	−129.4	−123.17	4.81	38.55	41.46	7.02

**Table 8:** Orthogonal Array L9($3^{4}$) Experimental Groups.

Sample	$n_{c}$ (r/min)	$f_{z}$ (mm/z)	$a_{p}$ (mm)	$a_{e}$ (mm)
1	3000	0.01	0.5	6
2	3000	0.02	1	9
3	3000	0.03	1.5	12
4	5000	0.01	1	12
5	5000	0.02	1.5	6
6	5000	0.03	0.5	9
7	7000	0.01	1.5	9
8	7000	0.02	0.5	12
9	7000	0.03	1	6

**Table 9:** Results of Orthogonal Experiment.

Sample	$\sigma_{X}$ (MPa)	$\sigma_{Y}$ (MPa)	$F_{X}$ (N)	$F_{Y}$ (N)
1	−51.41	−111.72	7.19	17.86
2	−37.63	−82.60	22.41	28.89
3	−18.24	−44.41	50.38	61.47
4	−41.60	−108.83	20.49	25.94
5	−39.81	−68.06	38.77	45.70
6	−34.23	−40.21	23.81	32.86
7	−38.84	−129.40	30.79	38.55
8	−24.31	−44.62	17.95	23.76
9	−37.96	−57.91	29.48	33.57

**Table 10:** Error analysis of surface residual stress empirical model.

$n_{c}$ (r/min)	$f_{z}$ (mm/z)	$a_{p}$ (mm)	$a_{e}$ (mm)	Pred. $\sigma_{X-p}$ (MPa)	Exp. $\sigma_{X-e}$ (MPa)	X Error (%)	Pred. $\sigma_{Y-p}$ (MPa)	Exp. $\sigma_{Y-e}$ (MPa)	Y Error (%)
3500	0.03	1	8	−29.55	−25.60	15.43	−49.73	−41.91	18.69
4500	0.02	1.5	10	−28.81	−31.92	9.74	−69.61	−62.53	11.38
5500	0.01	0.5	10	−42.03	−49.73	15.48	−94.49	−115.8	18.40

**Table 11:** Error analysis of the empirical cutting force model.

$n_{c}$ (r/min)	$f_{z}$ (mm/z)	$a_{p}$ (mm)	$a_{e}$ (mm)	Pred. $F_{X-p}$ (MPa)	Exp. $F_{X-e}$ (MPa)	X Error (%)	Pred. $F_{Y-p}$ (MPa)	Exp. $F_{Y-e}$ (MPa)	Y Error (%)
3500	0.03	1	8	31.41	33.03	4.9	40.74	36.18	11.19
4500	0.02	1.5	10	41.18	44.71	7.91	45.81	50.12	9.42
5500	0.01	0.5	10	10.79	11.86	9.01	18.45	20.71	12.27

**Table 12:** Range analysis of residual stress and cutting forces.

Response Type	Direction	Parameters	K1	K2	K3	k1	k2	k3	Range (R)	Ranking
residual stress	X	$n_{c}$	−107.28	−115.64	−101.11	−35.76	−38.55	−33.70	4.84	$a_{e}$ $\;>\;f_{z}$ $\;>\;a_{p}$ $\;>\;n_{c}$
		$f_{z}$	−131.85	−101.75	−90.43	−43.95	−33.92	−30.14	13.81	
		$a_{p}$	−109.95	−117.19	−96.89	−36.65	−39.06	−32.30	6.77	
		$a_{e}$	−129.18	−110.70	−84.15	−43.06	−36.90	−28.05	15.01	
	Y	$n_{c}$	−238.73	−217.10	−231.93	−79.58	−72.37	−77.31	7.21	$f_{z}$ $\;>\;a_{e}$ $\;>\;a_{p}$ $\;>\;n_{c}$
		$f_{z}$	−349.95	−195.28	−142.53	−116.65	−65.09	−47.51	69.14	
		$a_{p}$	−196.55	−249.34	−241.87	−65.52	−83.11	−80.62	17.60	
		$a_{e}$	−237.69	−252.21	−197.86	−79.23	−84.07	−65.95	18.12	
cutting force	X	$n_{c}$	81.14	83.07	78.22	27.05	27.69	26.07	1.62	$a_{p}$ $\;>\;f_{z}$ $\;>\;a_{e}$ $\;>\;n_{c}$
		$f_{z}$	58.47	80.29	103.67	19.49	26.76	34.56	15.07	
		$a_{p}$	48.95	73.54	119.94	16.32	24.51	39.98	23.66	
		$a_{e}$	75.44	78.17	88.82	25.15	26.06	29.61	4.46	
	Y	$n_{c}$	108.22	104.50	95.88	36.07	34.83	31.96	4.11	$a_{p}$ $\;>\;f_{z}$ $\;>\;a_{e}$ $\;>\;n_{c}$
		$f_{z}$	82.35	98.35	127.90	27.45	32.78	42.63	15.18	
		$a_{p}$	74.48	88.40	145.72	24.83	29.47	48.57	23.74	
		$a_{e}$	97.13	100.30	111.17	32.38	33.43	37.06	4.68	

## Data Availability

The original contributions presented in this study are included in the article. Further inquiries can be directed to the corresponding author.

## Appendix A. Codes/Acronyms

**Codes**	**Name**	**Acronyms**	**Name**
$\sigma_{s}$	Yield strength	Sim.	Simulate
$\sigma_{b}$	Tensile strength	Exp.	Experimental
ρ	Density	Pred.	Prediction
*E*	Elastic modulus	EDC	Exponential Decay Cosine
δ	Elongation	PSO	Particle Swarm Optimization
		HBW	Hardness
		BP	Backpropagation
